# A Copper Silicate-Based Multifunctional Nanoplatform with Glutathione Depletion and Hypoxia Relief for Synergistic Photodynamic/Chemodynamic Therapy

**DOI:** 10.3390/ma17143495

**Published:** 2024-07-15

**Authors:** Meiqi Shao, Wei Zhang, Fu Wang, Lan Wang, Hong Du

**Affiliations:** 1 Xinjiang Key Laboratory of Energy Storage and Photoelectrocatalytic Materials & Chemistry and Chemical Engineering, Xinjiang Normal University, Urumqi 830054, China; meiqishao254@163.com; 2 Shenzhen Research Institute, Shanghai Jiao Tong University, Shenzhen 518057, China; wangfu@sjtu.edu.cn; 3 Environmental Science and Engineering, Shaanxi University of Science and Technology, Xi’an 710021, China; zhangwei149817@163.com

**Keywords:** chemodynamic therapy, photodynamic therapy, synergistic therapy, layered copper silicate, reactive oxygen species, antitumor, tumor hypoxia

## Abstract

Chemodynamic therapy (CDT) alone cannot achieve sufficient therapeutic effects due to the excessive glutathione (GSH) and hypoxia in the tumor microenvironment (TME). Developing a novel strategy to improve efficiency is urgently needed. Herein, we prepared a copper silicate nanoplatform (CSNP) derived from colloidal silica. The Cu(II) in CSNP can be reduced to Cu(I), which cascades to induce a subsequent CDT process. Additionally, benefiting from GSH depletion and oxygen (O_2_) generation under 660 nm laser irradiation, CSNP exhibits both Fenton-like and hypoxia-alleviating activities, contributing to the effective generation of superoxide anion radical (^•^O_2_^−^) and hydroxyl radical (^•^OH) in the TME. Furthermore, given the suitable band-gap characteristic and excellent photochemical properties, CSNP can also serve as an efficient type-I photosensitizer for photodynamic therapy (PDT). The synergistic CDT/PDT activity of CSNP presents an efficient antitumor effect and biosecurity in both in vitro and in vivo experiments. The development of an all-in-one nanoplatform that integrates Fenton-like and photosensing properties could improve ROS production within tumors. This study highlights the potential of silicate nanomaterials in cancer treatment.

## 1. Introduction

Cancer is one of the most intricate diseases posing a significant threat to people, profoundly impacting the quality of human health [1,2]. As a strategy for attacking tumors, reactive oxygen species (ROS)-based therapy, using, e.g., superoxide anion (^•^O_2_^−^), hydrogen peroxide (H_2_O_2_), and hydroxyl radical (^•^OH), possesses the capability to induce cell apoptosis or necrosis through approaches such as chemodynamic therapy (CDT) [3], photodynamic therapy (PDT) [4], and sonodynamic therapy (SDT) [5]. Specifically, CDT is a promising and effective tumor treatment method developed rapidly in recent years [6,7,8]. The metal ions catalyze endogenous H_2_O_2_ through the Fenton reaction to form ^•^OH, which is the most harmful radical in the ROS. However, generating sufficient ROS at specific tumor sites remains challenging. Considering the complexity of tumor treatment, monotherapy is less effective in treating cancers, so there is an urgent need to develop synergistic approaches to enhance efficacy and reduce toxic side effects for treatment. Various synergistic therapies such as CDT/PDT, CDT/PTT, and CDT/immunotherapy hold promise for enhanced therapeutic efficacy through the utilization of synergistic mechanisms [9,10,11].

Copper (Cu) serves as a vital element for all living organisms, and is recognized as a catalyst for Fenton-like reactions [12]. Cu(II) can deplete overexpressed glutathione (GSH) in tumor cells and turn into Cu(I) to trigger the Fenton-like reaction, producing cytotoxic ^•^OH for cancer treatment [13]. However, low levels of H_2_O_2_ (100 μM) and a weakly acidic tumor microenvironment (TME) (pH 6.5–7.0) can impede the efficiency of the Fenton-like reaction [14,15]. As one of the emerging therapy modalities, PDT generates high levels of ROS through the photoactivation of a photosensitizer (ps); specifically, near-infrared (NIR) light-triggered PS has gained much attention [16,17,18]. However, the progression of PDT is limited by the hypoxia TME, and traditional organic photosensitizers generally lack photostability [19,20]. In recent years, continuous efforts have been committed to integrate Fenton/Fenton-like agents with photosensitizers into a multicomponent nanoplatform, such as a HA/CaO_2_-Ce6@Cu-ZIF nanoplatform [21] or Cu/CaCO_3_@Ce6 nanoparticles [22]. Previous synergistic CDT/PDT strategies have primarily involved the load of Fenton or Fenton-like catalysts and photosensitizers to achieve an integrated multifunctionality. Nevertheless, the realization of synergistic CDT/PDT therapy within a single nanoplatform remains rare. Recently, Liu et al. synthesized mesoporous copper/manganese silicate nanospheres coated with cancer cell membranes (mCMSNs) for CDT/PDT synergistic therapy with a 635 nm laser. However, the theranostic nanoagents are limited in synergistic CDT/PDT effects and biocompatibility [23,24,25].

Silicate-based nanomaterials, characterized by a large surface-to-volume ratio, tunable bandgap structures, and good near-infrared light responsiveness, have been employed in biomedical applications, wound healing, epithelialization, and collagen deposition [26,27]. Utilizing the inherent structural and compositional advantages of natural layered minerals, researchers have incorporated transition metals into the octahedral sites of synthesized layered silicates [28,29,30]. However, manganese ions (Mn^2+^) exhibit Fenton-like activity exclusively in the presence of bicarbonate ions (HCO_3_^−^) [31,32], while iron ions (Fe^2+^) demonstrate a comparatively slower Fenton reaction rate, even in more acidic environments [33,34]. So it is desirable to develop a transition metal nanoplatform that is conducive to a Fenton-like reaction with the assistant of near-infrared (NIR) PDT performance [35].

**Figure 1:**
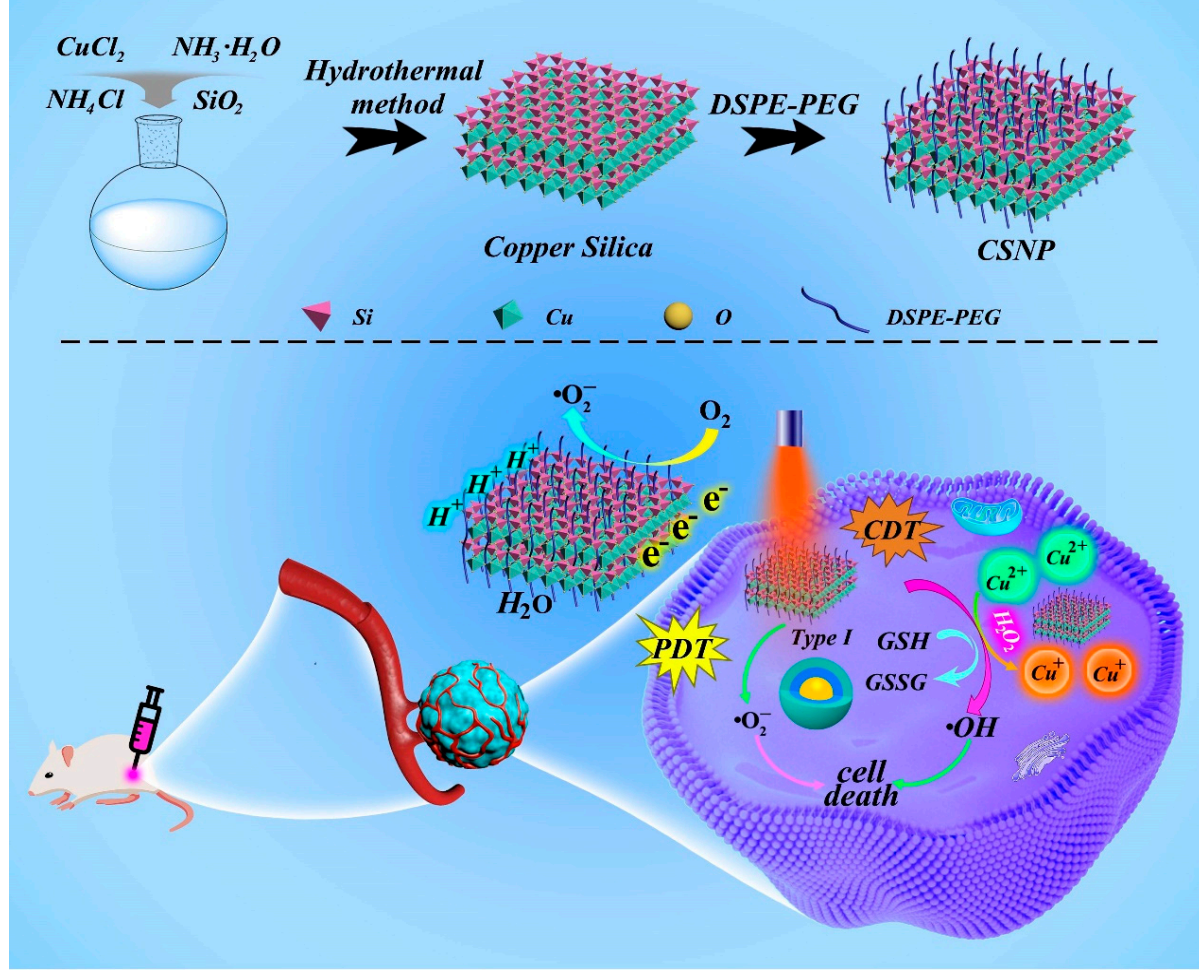
Therapeutic mechanism of CSNP for PDT under laser with tumor hypoxia modulation and GSH-triggered CDT.

In this study, we synthesized a biocompatible copper silicate nanoplatform using the hydrothermal method. The CSNP modified with DSPE-PEG2000 exhibited good dispersion in physiological environments. As a Fenton-like catalyst, the Cu(II)/Cu(I) redox couple endowed the CSNP with ^•^OH generation capability. By means of a redox reaction, the Cu(II) ions in CSNP overexpressed GSH in the TME. Then, the generated Cu(I) converted endogenous H_2_O_2_ into toxic ^•^OH via the Fenton-like reaction. Acting as an effective photosensitizer for PDT, CSNP has a relatively narrow band gap of 2.64 eV, and the bandgap edges of CSNP can match the redox potentials of O_2_/^•^O_2_^−^ and O_2_/H_2_O. Photogenerated electrons and holes (e_CB_^−^/h_VB_^+^) can generate cytotoxic ^•^O_2_^−^ and simultaneously catalyze the splitting of water to produce O_2_ (Figure 1). Therefore, CSNP is a promising nanomaterial which shows the combination of CDT and PDT in the synergistic treatment of cancer.

## 2. Materials and Methods

### 2.1. Materials

Colloidal silica (LUDOX AS-40, JSENB, Shenzhen, China, 40 wt% suspension in H_2_O), cupric chloride dihydrate (CuCl_2_·2H_2_O, AR), aqueous ammonia (NH_3_·H_2_O, 25–28%), and ammonium chloride (NH_4_Cl, 99.5%) were purchased from Aladdin Industrial Cooperation, Shanghai, China. All reagents were not purified. 5,5′-dithiobis(2-nitrobenzoic-acid) (DTNB), 3,3′,5,5′-tetramethylbenzidine (TMB), and 1,3-diphenylisobenzofuran (DPBF) were obtained from Aladdin Industrial Cooperation, Shanghai, China. DSPE-PEG2000 were purchased from Xi’an Qiyue Biotechnology Co., Ltd. (Xi’an, China). Dihydroethidium (DHE), 2′,7′-dichlorofluorescin diacetate (DCFH-DA), and calcein-AM/propidium iodide (PI) were obtained from Sigma-Aldrich (St. Louis, MO, USA). 3-[4,5-dimethylthiazol-2-yl]-2,5-diphenyltetrazolium bromide (MTT) was from Beyotime (Beyotime Biotechnology, Shanghai, China).

### 2.2. Instruments

UV-vis-NIR spectra were monitored using a UV-1800 (Shimadzu, Tokyo, Japan) spectrophotometer. The fluorescence spectrophotometer (RF-6000, Shimadzu, Kyoto, Japan) measured the PL spectrum. All cell photographs were captured with confocal laser scanning microscopy (CLSM, Leica TCS SP8, Wetzlar, Germany). Nanoplatform size and zeta potential were measured using dynamic light scattering (DLS) (Brookhaven ZetaPALS, Brookhaven Instruments Corporation, Holtsville, NY, USA). Transmission electron microscopy (TEM) and energy dispersive spectrometry (EDS) elemental mapping images were obtained from a Talos F200X device (Thermo Fisher Scientific, Waltham, MA, USA). X-ray diffraction (XRD) was conducted with an X-ray diffractometer (Empyrean, Malvern Panalytical, Almelo, The Netherlands) using a Cu Kα X-ray source (λ = 1.5406 Å). X-ray photoelectron spectroscopy (XPS) was carried out via an Axis-Ultra DLD (Shimadzu, Tokyo, Japan) equipped with a monochromatic X-ray source (Al Kα, hυ = 1486.6 eV). The Mott–Schottky measurement was carried out on a potentiostat/galvanostat (CHI660E, Shanghai Chenhua Co., Shanghai, China). A 660 nm laser was purchased from Changchun Institute of Optics, Fine Mechanics & Physics, Chinese Academy of Sciences (Changchun, China).

### 2.3. Preparation of Copper Silicate

In a typical procedure, cupric chloride dihydrate (0.75 mM) was dissolved in 50 mL of deionized water. Then, aqueous ammonia (3 mL, 25 wt%), colloidal silica (LUDOX AS-40, 0.64 mL), and ammonium chloride (10 mM) were added to the above mixture, and then the solution was removed into a hydrothermal autoclave (100 mL) followed by heating at 140 °C for 12 h. The reaction product was separated through centrifugation, washed three times with 30 mL of deionized water, and dried for 24 h under vacuum. Next, the prepared material (0.2 g) was dissolved in 40 mL of ultrapure water. The silica substrate with a CSNP attachment can be separated during centrifugation at lower rotational speeds (1500 r/min for 5 min). In a subsequent step, the remaining suspension was sonicated for 50 min in an ultrasonic cell crusher (BILON-250Y, Shanghai Bilon Instrument Co., Ltd., Shanghai, China) and allowed to stand for 6 h. Finally, the supernatant was dried in a vacuum oven at 60 °C for 24 h.

### 2.4. Synthesis of DSPE-PEG2000-Coated CSNP

DSPE-PEG2000 (16 mg) was dissolved in deionized water (20 mL) and subjected to sonication at 25 °C about 5 min, and then 8 mg of copper silicate powder was promptly added into the above solution and sonicated for 30 min. The prepared solution was centrifuged for 5 min at 5000 r/min and filtered through a 0.22 μM membrane filter to remove any residue. The solution was vortexed for further use.

### 2.5. GSH Depletion Ability of CSNP

Glutathione (GSH) reacts with 5,5′-dithiobis-2-nitrobenzoic acid (DTNB) to form the yellow 2-nitro-5-thiobenzoate (TNB). The DTNB reacting with GSH showed an absorption peak around 412 nm. Briefly, a mixture was prepared containing reduced GSH (20 μL, 10 mM) and DTNB (30 μL, 10 mM) in PBS (0.1 M, pH = 6.5). Subsequently, varying CSNP concentrations (0–200 μg/mL) were added and the mixture was incubated for 5 min. Finally, the absorbance of the mixture was monitored using UV-vis-NIR absorption spectroscopy.

### 2.6. ^•^OH Production by TMB

To evaluate the ^•^OH generation capacity of the CSNP, TMB can be oxidized by ^•^OH into oxTMB (blue). In detail, CSNP (2 mL, 400 μg/mL) at pH 5.0, 6.5 or 7.4 and GSH (30 μL, 10 mM) were shaken for 30 min. Then, H_2_O_2_ (20 μL, 1 mM) and TMB solution (30 μL, 1 mg/mL) was added into the mixture solutions followed by incubation for 45 min. Lastly, the UV absorbance of TMB at 652 nm was measured on a UV-vis-NIR spectrometer.

### 2.7. ^•^O_2_^−^ Detection by DPBF

1,3-diphenylisobenzofuran (DPBF) was used to detect ^•^O_2_^−^ with the absorbance change at 412 nm. Under normoxic conditions, CSNP solution (2 mL, 400 μg/mL) containing DPBF (30 μL, 1 mg/mL) was irradiated with a 660 nm laser (1.0 W·cm^−2^). UV-vis-NIR spectra were recorded at 2 min intervals over a period of 10 min during the testing of the solution. To detect ^•^O_2_^−^ generation under hypoxic conditions, another reaction solution was degassed by bubbling argon gas for 10 min and was tested using the same method as previously described.

### 2.8. Total ROS Detection

2′,7′-dichlorofluorescein (DCFH) was utilized as a probe to assess total ROS levels. The CSNP solution (2 mL, 400 μg/mL) containing 50 µM DCFH was irradiated with a 660 nm laser (1.0 W cm^−2^). Fluorescence emission at 525 nm was recorded at a 488 nm excitation wavelength every 30 s. A sample of the DCFH mixture without CSNP was treated in the same way for comparison.

### 2.9. Cell Culture

Mouse 293T cells (ATCC No CRL-3216) and CT26 cells (ATCC No CRL-2638) were obtained from Sangon Biotech (Shanghai, China). During routine culture, the 293T cells were cultured in DMEM medium, while the CT26 cells were cultured in 1640 medium. These cell lines were cultured in a cell culture incubator at 37 °C, 5% CO_2_, and 95% humidity.

### 2.10. In Vitro Cytotoxicity

The 293T cells and CT26 cells were seeded in 96-well plates for 24 h. Subsequently, the medium was exchanged with new medium containing varying concentrations of CSNP (0–80 μg/mL) in each well, and the cells were further incubated for 12 h. To assess the dark toxicity of CSNP on 293T cells and CT26 cells, the cells were cultured without laser treatment. Then, the culture medium in the CT26 cells (80 μg/mL) was replaced by the new culture medium containing H_2_O_2_ (100 μM) at pH = 6.5. The cells were irradiated with or without a 660 nm laser (1.0 W·cm^−2^) for 5 min. After 12 h incubation, 20 μL of MTT (5 mg/mL) was added and incubated for 4 h in the incubator. Finally, the supernatant was discarded and 150 μL DMSO was added for the cytotoxicity assay. The absorbance of all wells was tested using a microplate reader at a wavelength of 490 nm. Furthermore, the cytotoxicity of CSNP on the CT26 cells was further checked by means of Calcein-AM and propidium iodide (PI) staining. The CT26 cells were cultured in 6-well plates while being divided into different groups (Dark, Laser, H_2_O_2_, CSNP, CSNP + H_2_O_2_, CSNP + L, and CSNP + H_2_O_2_ + L). The appropriate wells were incubated in the presence of 30 μg/mL of CSNP. After treatment with or without a 660 nm laser (1.0 W cm^−2^, 10 min), each plate was stained with Calcein-AM and propidium iodide (PI) for 30 min. Live cells were stained with Calcein-AM, while dead cells were stained with propidium iodide. Then, the cells were fixed with 4% paraformaldehyde, and confocal laser scanning microscopy (CLSM) was employed to capture the fluorescence images of the cells.

### 2.11. Cellular Uptake Behavior

Under dark conditions, Ce6-PEG2000 (10 mg) was dissolved in water (5 mL) to a final concentration of 2 mg/mL. Subsequently, CSNP (2 mg) was quickly added to the above homogeneous solution and sonicated for half an hour to reach a homogeneous dispersion. CT26 cells were cultured with various concentrations of PEG-Ce6-coated CSNP (10, 30, 50, and 80 μg/mL) in 6-well plates for 12 h. Next, the cells were washed twice with PBS and fixed with 4% paraformaldehyde for 20 min. The images were obtained using a confocal laser scanning microscope (CLSM).

### 2.12. Hemolysis Assay

Eyeball whole blood was collected from BALB/c mice and injected in ethylenediamine tetraacetic acid (EDTA) tubes. The fresh blood was washed three times with saline. After centrifugation at 3000 rpm/min for 10 min, the red blood cell suspension was collected and incubated with normal saline, water, and different concentrations of CSNP ranging from 20 to 150 μg/mL for 6 h. Finally, the absorption value of the supernatant was measured using a UV-vis-NIR spectrophotometer at 541 nm. To calculate the hemolysis rate (HR), the following formula was used: Hemolysis rate = (A sample − A negative)/(A positive − A negative).

### 2.13. In Vivo Synergistic Therapy

To evaluate the synergistic CDT/PDT effect of CSNP, BALB/c female mice were subjected to standard subcutaneous inoculation to establish a CT26 tumor model. BALB/c (5-week-old) mice were subcutaneously injected with CT26 tumor cells (1 × 10^6^) on right thigh. Once the tumor volume reached 100 mm^3^, the mice were randomly separated into three groups (n = 3 per group): PBS, CSNP (2 mg/kg), and CSNP (2 mg/kg) plus 660 nm irradiation (1 W cm^−2^, 5 min) for different treatment. The mice were intratumorally injected with different formulations every third day for a total of four doses. During the period of the 14-day treatment, tumor size was recorded with electronic calipers every 2 days. The mice were humanely killed on day 14 and their tumors were dissected, weighed, and photographed. The tumor volume was figured out using the following formula: Tumor volume (mm^3^) = length × width × width/2.

### 2.14. Toxicology Analysis

After completing the assessment of antitumor efficacy, all animals were humanely euthanized and major organs were harvested for further histological examination. Next, the organs (the heart, liver, spleen, lungs, and kidneys) were harvested and fixed with 4% paraformaldehyde. H&E staining was conducted for detecting the tumor cell damage. Moreover, blood samples were collected from tumor-bearing mice to assess liver and kidney functions.

## 3. Results and Discussion

### 3.1. Synthesis and Characterization of CSNP

**Figure 2:**
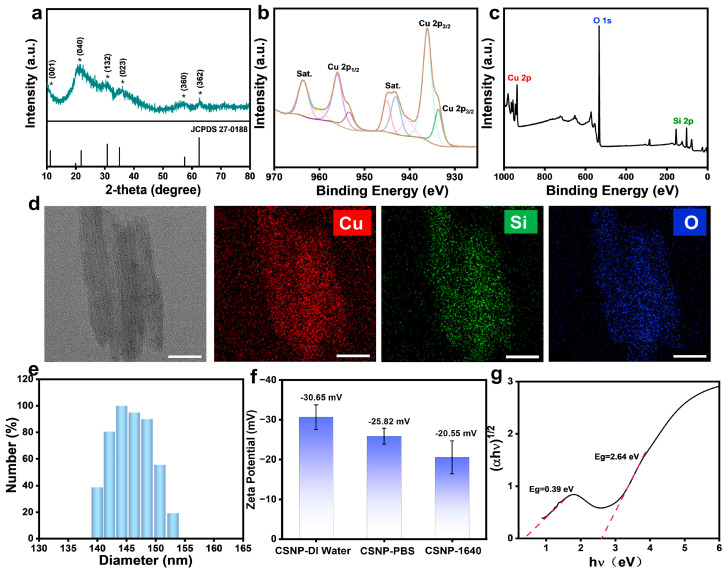
(**a**) Power XRD spectrum of CSNP nanoplatform (JCPDS 27-0188). (**b**) High-resolution XPS spectra of Cu 2p in CSNP. (**c**) Wide-scan XPS spectrum of CSNP. (**d**) HAADF-STEM image and elemental mapping of Cu, Si, O of CSNP. Scale bar: 50 nm. (**e**) Size distribution of CSNP by DLS in DI water. (**f**) Surface zeta potential of CSNP in DI Water, PBS, and RPMI-1640 medium. (**g**) Tauc plots of (αhv)^1/2^ vs. hv.

Using colloidal silica as raw material, we synthesized copper silicate using the hydrothermal method and then prepared a CSNP encapsulated by DSPE-PEG2000 to enhance water solubility and biocompatibility. The X-ray diffraction (XRD) pattern of CSNP is displayed in Figure 2a, and all diffraction peaks can be well indexed according to the structure of copper silicate Cu_2_Si_2_O_5_(OH)_2_ (JCPDS card no. 27-0188) [36,37]. As shown in Figure 2b, the peaks at 932.4 eV and 954.2 eV were attributed to Cu 2p_3/2_ and Cu 2p_1/2_ of Cu^+^, while the binding energy peaks of 934.8 eV and 956.6 eV pointed to the Cu 2p_3/2_ and Cu 2p_1/2_ peaks of Cu^2+^, respectively. This result confirmed that Cu has two valence states (Cu^+^ and Cu^2+^) in CSNP. The Si 2p peak was found at 102.5 eV (Figure S2a of Supplementary Materials), close to the reported similar binding energy values of silicate [38]. The X-ray photoelectron spectroscopy (XPS) spectrum also showed the presence of Cu, Si, and O elements in the CSNP (Figure 2c). The High-Angle Angular Dark-Field Scanning Transmission Electron Microscopy (HAADF-STEM) images exhibited the layered material of CSNP with a diameter of 158 nm (Figure 2d). And the energy dispersive spectrometer (EDS)-elemental mappings were used to investigate the elemental distribution (Figure S2b), which further confirmed the successful synthesis. The effective diameter of CSNP in water (pH 6.5), measured with the DLS test (Figure 2e), was 145 nm, which was consistent with the HAADF-STEM images. The nanostructure CSNP possessed surface zeta potentials of −30.65 mV, −25.82 mV, and −20.55 mV in water and PBS and 1640 media, respectively (Figure 2f). These results confirmed that copper silicate was successfully synthesized.

**Figure 3:**
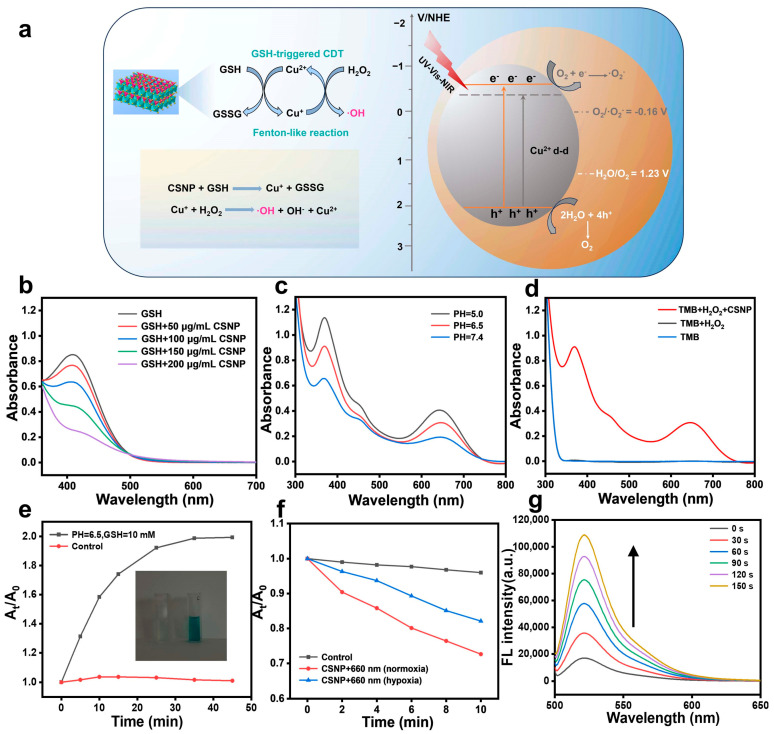
(**a**) Schematic diagram of the CDT process of CSNP as a natural Fenton-like nanoplatform and PDT process of electron/hole separation and photo-excited reaction of CSNP (NHE = normal hydrogen electrode); (**b**) GSH depletion curves of CSNP at different concentrations. (**c**) UV-vis-NIR spectrum of TMB solution containing CSNP and H_2_O_2_ at different pHs. (**d**) UV-vis-NIR spectrum of TMB solution (pH = 6.5) under different conditions (None, H_2_O_2_, and H_2_O_2_ + CSNP). (**e**) Comparison of CDT performance of without or with GSH. (**f**) Comparison of PDT performance of normoxia or hypoxia condition. (**g**) The fluorescence intensity of the solution with DCFH and CSNP under different 660 nm irradiation time.

The UV-vis-NIR diffuse reflectance spectrum of thesynthesized CSNP was used to clarify the optical absorption property and energy bandgap (Figure 2g). The indirect band gap value extracted from Tauc plots is 2.64 eV and the broad long-wavelength NIR absorption is attributed to the d-d transition of Cu^2+^ (Figure S3a). The CB value of CSNP was estimated to be −0.393 eV (vs. NHE) based on the Mott–Schottky (Figure S3b). The VB position was further estimated through the CB value and the band gap value, which was estimated to be 2.247 eV. Therefore, as shown in Figure 3a, CSNP can react with GSH to generate Cu^+^ for the CDT process, in which GSH is oxidized to GSSG. Based upon the energy band diagram of the material, the potentials of O_2_/^•^O_2_^−^ and H_2_O/O_2_ implied that the CSNP can generate ^•^O_2_^−^ and realize O_2_ evolution simultaneously under NIR exposure. The synergistic CDT/PDT system can enhance ROS generation for cancer treatment.

### 3.2. GSH Depletion and CDT/PDT Performance Tests

As shown in Figure 3b, the GSH depletion capacity of CSNP was demonstrated by the absorbance changes of 5,5′-dithiobis-(2-nitrobenzoic acid) (DTNB) [39]. An absorbance at 412 nm showed a significant decrease with the increase in CSNP concentration, indicating a redox reaction between GSH and Cu(II), resulting in continuous GSH depletion. To evaluate the ^•^OH generation of CSNP through a Fenton-like reaction, a 3,3′,5,5′-tetramethylbenzidine (TMB) probe was employed as an indicator [40]. The generated Cu(I) readily reacts with H_2_O_2_ to produce ^•^OH. CSNP was added to the mixed solution of TMB and H_2_O_2_ at different pHs (Figure 3c). The characteristic absorption of TMB at 340 and 650 nm increased as the pH decreased from 7.4 to 5.0, indicating that the degradation efficiency was highest at pH = 5.0. It is evident that low pH values intensified the Fenton-like reaction. Given that the TME was characterized by low acidity (generally around 6.5), CSNP also exhibited strong catalytic activity. When TMB was mixed with either CSNP or H_2_O_2_ alone under mild acidity (pH = 6.5), no obvious absorption peak could be observed in Figure 3d. After adding CSNP and H_2_O_2_ at the same time, a strong absorption peak of oxTMB was found at 370 and 652 nm, indicating that CSNP effectively generated ^•^OH through the catalytic reaction of H_2_O_2_. The absorption peak intensity of the mixture containing TMB and CSNP maintained a continuous increase within 45 min incubation with GSH (10 mM) and H_2_O_2_ (1 mM) at pH = 6.5. In contrast, control treatment showed no significant change in TMB absorbance (Figure 3e and Figure S4a,b). Additionally, the TMB solution clearly displayed a color change from colorless to blue, further demonstrating ^•^OH generation ability. Then, ^•^O_2_^−^ detection was determined using 1,3-diphenylisobenzofuran (DPBF) as a detection probe [41]. The groups of the CSNP plus 660 nm in the normoxic and hypoxic condition are shown in Figure 3f; the absorbance of DPBF at 412 nm decreased gradually with prolonged 660 nm laser irradiation time, which can be attributed to the generation of ROS and O_2_ by the photoinduced electron (e_CB_^−^) and hole (h_VB_^+^) of the CSNP (Figure S4c,d). Moreover, total ROS was estimated using a 2′,7′-Dichlorofluorescein (DCFH) probe. As exhibited in Figure 3g, the DCF fluorescence signal in the CSNP solution significantly increased under a 660 nm laser irradiation, while a subtle change in DCF fluorescence in the aqueous solution without CSNP was detected in the absence of exogenous oxidants (Figure S2c).

### 3.3. In Vitro Cytotoxicity

**Figure 4:**
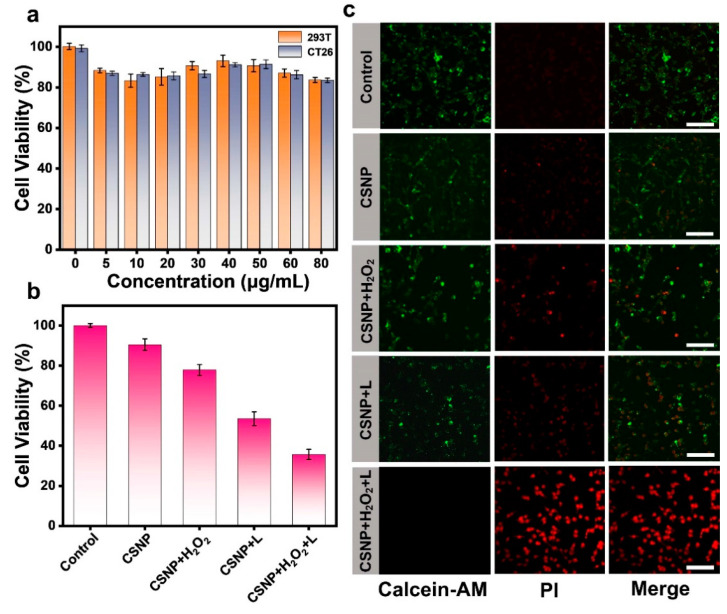
(**a**) Cell viability of 293T and CT26 cells treated with varied concentrations of CSNP without laser exposure. (**b**) Phototoxicity of CSNP toward CT26 cells with different treatments. (**c**) The pictures of CT26 cells co-stained Calcein-AM/PI under different treatments by CLSM. Scale bar: 50 μm.

The cytotoxicity of CSNP was assessed by means of a standard 3-(4,5-dimethylthiazol-2-yl)-2,5-diphenyltetrazolium bromide (MTT) assay. As shown in Figure 4a, 293T cells and CT26 cells were incubated with various concentrations of CSNP for 24 h, displaying no significant toxicity even at the highest concentrations of CSNP. The high cell viability observed in the presence of only light and H_2_O_2_ indicated that neither has an effect on the cells (Figure S5). However, when the CT26 cells were incubated with CSNP (80 μg/mL) and H_2_O_2_ (100 μM) for 24 h, cell viability was slightly decreased. This revealed that H_2_O_2_ can be converted to cytotoxic ^•^OH via the Fenton-like reaction. Next, CT26 cell viability following treatment with the same concentration of CSNP and exposure to 660 nm laser irradiation showed similarly low survival rates. When it comes to collaborative therapy, the CT26 cells incubated with CSNP and further treated with H_2_O_2_ and 660 nm laser irradiation showed a precipitous decline, demonstrating the outstanding enhanced efficiency of CDT and PDT (Figure 4b). In addition, cell killing by CDT/PDT was accessed using Calcein-AM (live cells; green) and propidium iodide (dead cells; red) staining (Figure 4c). Laser- and H_2_O_2_-only-treated cells exhibited no red fluorescence (dead cells) (Figure S6). Compared with control, the single CSNP plus H_2_O_2_ group or CSNP plus 660 nm laser irradiation group showed small amounts of red fluorescence signals. When CSNP treated with H_2_O_2_ was exposed to 660 nm laser irradiation, the most significantly strong PI fluorescence (red) was observed. These results demonstrate that the cancer cell killing ability of CDT/PDT was superior than that of CDT or PDT. Overall, CSNP can be used as an effective photosensitizer and Fenton-like agent for further study.

### 3.4. In Vitro Cellular Uptake and Intracellular ROS Detection

**Figure 5:**
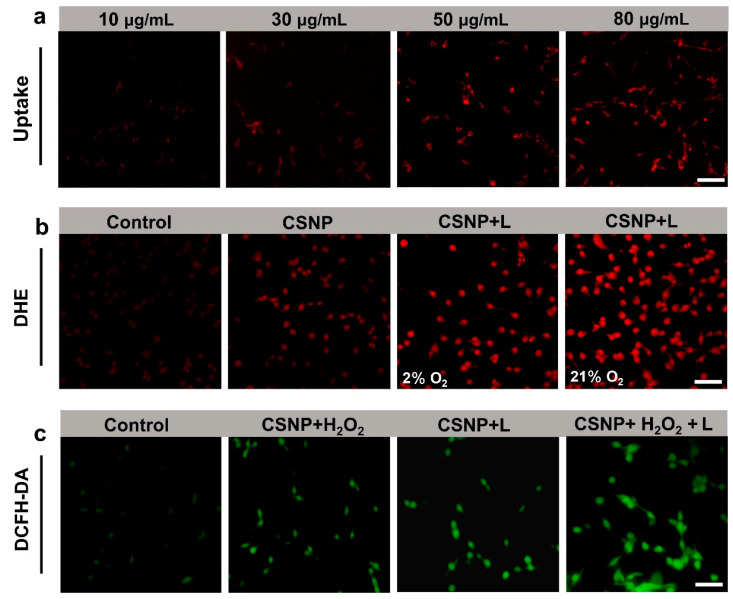
(**a**) Cellular uptake of PEG-Ce6-coated CSNP at different concentrations. (**b**) Detection of ^•^O_2_^−^ in CT26 cells with DHE probe under hypoxia (2% O_2_) or normoxia (21% O_2_) conditions. (**c**) Fluorescence images of ROS production in CT26 cells stained with DCFH-DA (a green ROS probe) following different treatments. Scale bar: 50 μm.

Considering that the CSNP lacked fluorescent signals, we used PEG-Ce6 to wrap the CSNP and subsequently incubated them with CT26 cells. As shown in Figure 5a, confocal images showed that the red fluorescent signal in the cytoplasm gradually increased from 10 μg/mL to 80 μg/mL, indicating the effective endocytosis and accumulation behavior of CSNP. Based on the good ROS generation in the aqueous solution of CSNP, DHE can be oxidized by ^•^O_2_^−^ into ethidium bromide, intercalating into the DNA and generating a strong red fluorescence [42]. As shown in Figure S7a, CT26 cells treated with H_2_O_2_ exhibited almost no production of ^•^O_2_^−^. As shown in Figure 5b, compared with control and the CSNP-only group, the red fluorescence of the laser irradiation was getting stronger, proving that CSNP could generate cytotoxic radicals upon 660 nm laser irradiation in normoxia and hypoxia. The ^•^O_2_^−^ generation in hypoxia can be ascribed to the oxygen self-supply of CSNP upon 660 nm laser irradiation. Furthermore, a fluorescent probe of 2′,7′-Dichlorofluorescin diacetate (DCFH-DA) was used to examine the intracellular ROS generation ability of CSNP (Figure 5c) [43]. DCFH-DA was hydrolyzed by intracellular esterase to produce DCFH, and weak fluorescent DCFH was oxidized by ROS to produce strong green fluorescent DCF. When compared with the control, H_2_O_2_-only, and CSNP-only groups (Figure S7b), there was almost no green fluorescence. In the comparison between the CSNP plus H_2_O_2_ group and CSNP plus 660 nm laser group, a weak green fluorescent signal was observed, which suggested the generation of a small amount of ^•^OH or ^•^O_2_^−^. After CSNP treatment with H_2_O_2_ and 660 nm laser irradiation, the CT26 cells showed the strongest fluorescence signal (green), demonstrating that the CDT/PDT synergy effect enhanced ROS generation. Thus, these results demonstrated the advantage of the synergistic effect of CDT and PDT over monotherapy in tumor treatment.

### 3.5. Synergistic CDT/PDT Antitumor Effects in Vivo and Biochemistry Evaluation

**Figure 6:**
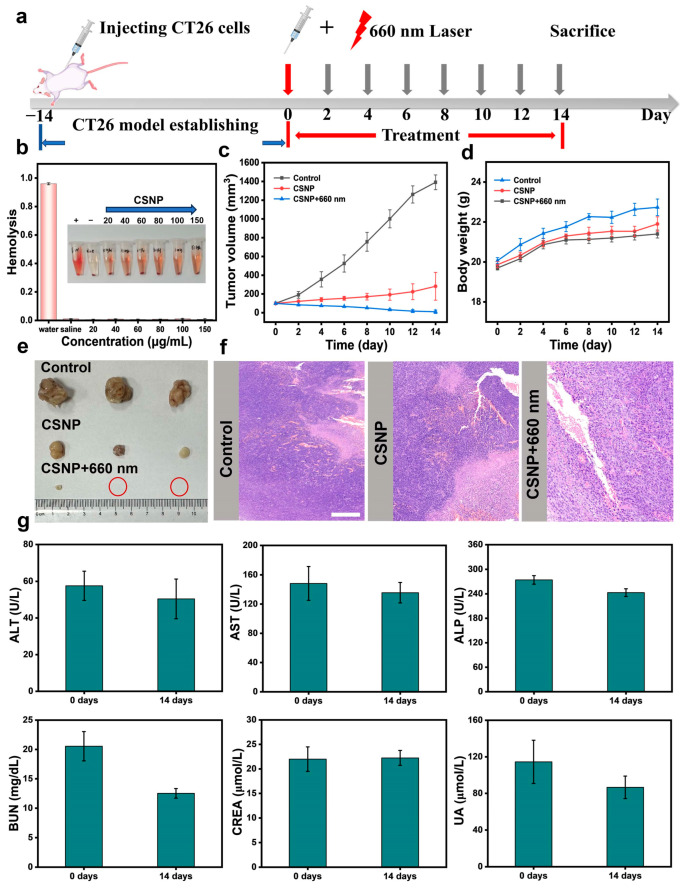
(**a**) Schematic illustration of the synergistic PDT and CDT regimen in a colorectal cancer mouse model. (**b**) Hemolysis rate at different concentrations of CSNP using saline and water as the negative and positive controls. (**c**) The growth curve of the tumor volume after different treatments. (**d**) Body weight change of CT26-bearing mice in the different treatments during 14 days. (**e**) Photographs of tumors dissected from living mice after various treatments. (**f**) Representative pictures of tumor tissue stained with hematoxylin-eosin from each group. Scale bar: 100 μm. (**g**) Blood analysis data of the CT26-bearing mice following an intratumoral injection of CSNP on Days 0 and 14 (CSNP at 10 mg/kg/dose; mean ± SD).

We performed the hemolysis test to validate the biosafety of CSNP in vivo. The hemolysis rate of different concentrations of CSNP was less than 4% (Figure 6b), demonstrating the good biocompatibility of CSNP. As shown in Figure 6a, motivated by the excellent CDT and PDT effect of CSNP in vitro, a CT26 tumor-bearing BALB/c mouse model (female, 5 weeks old) was further chosen for in vivo evaluation. Mice were randomly divided into three groups (n = 3 per group): (a) control group with saline, (b) intratumoral injection with CSNP (CDT group), and (c) intratumoral injection with CSNP plus 660 nm laser irradiation (CDT/PDT group). Mice injected with PBS exhibited rapid tumor growth, as shown in Figure 6c. By contrast, the other two groups exhibited delayed tumor growth. Compared with the single CDT group, mice treated with the CSNP plus 660 nm laser irradiation showed better therapeutic effect within 14 days, proving that the CSNP possessed excellent antitumor efficacy. Moreover, there were no obvious differences in mouse body weight during the observation period (Figure 6d). After 14 days, the image of tumors isolated from the sacrificed BALB/c mice intuitively displayed that the solid tumor of the c group significantly shrank, demonstrating the good therapeutic effect in comparison with the a and b groups (Figure 6e). The above results suggested that the synergistic CDT/PDT therapies exhibited great antitumor efficacy. The consequence of synergistic CDT and PDT was also examined using hematoxylin and eosin (H&E) staining (Figure 6f). It can be seen that the CDT-only group and the CDT/PDT group showed various degrees of damage compared with the saline group. The organs of mice (heart, liver, spleen, lungs, and kidneys) were subjected to H&E staining (Figure S8). No distinctly noticeable abnormality was observed in these organs, implying a negligible systemic toxicity of CSNP. All tested hepatic and renal indices of mice showed no obvious changes at 0 and 14 days post intratumoral injection including liver function markers ALT, AST, and ALP, and kidney function markers BUN, CRE, and UA. (Figure 6g) [44,45]. These results demonstrated that liver and kidney function indicators were all within the normal levels. Hence, the CSNP was a safe nanoplatform for synergistic CDT/PDT combination therapy.

The outstanding antitumor activity is benefited from the chemodynamic and photodynamic synergistic therapy [46,47]. PDT is an FDA-approved method for treatment [48], and the relative long wavelength light can increase the penetration depth of the treatment. However, the traditional PDT treatment method using 660 nm excitation light still has certain limitations such as insufficient tissue penetration [49,50]. In future work, due to the presence of Cu ions, it is expected to explore the role of the nanoplatform in disease diagnosis. Cu ions can act as a contrast agent for MRI [51]. In addition, Cu^2+^/Cu^+^ ions act via Fenton-like reactions to deplete glutathione and generate cytotoxic ^•^OH. The generation of ^•^O_2_^−^ under 660 nm laser indicates that the CSNP can achieve PDT even under hypoxic conditions. Intracellular ROS generation was measured using a fluorescent probe DCFH-DA. In brief, the synthesized multifunctional copper silica nanoplatform displayed great potential for synergistic CDT/PDT. The efficacy of this combined treatment was demonstrated in mouse colorectal cancer models.

## 4. Conclusions

In conclusion, a copper silicate nanoplatform was synthesized with the hydrothermal method using colloidal silica as silicon template for synergistic CDT/PDT therapy. After being encapsulated by DSPE-PEG2000 (named CSNP), it showed good biocompatibility and low toxicity. Cu(II) was abundantly present in the CSNP, and depleted GSH in a weakly acidic environment to catalyze endogenous H_2_O_2_ to produce cytotoxic ^•^OH via a Fenton-like reaction. In addition, our results showed that the CSNP has unique electronic energy band structure for NIR-activated photocatalysis. As an inorganic photosensitize, the CSNP can generate cytotoxic ^•^O_2_^−^ and oxygen under 660 nm laser irradiation. In vitro experiments showed that CDT/PDT therapy is a better strategy for cancer treatment than monotherapy. Therefore, the combination of CDT and PDT synergistically improves antitumor efficacy against hypoxic solid tumors. This study provided a new avenue for monometallic silicate nanomaterials, as potential anticancer agents, for the treatment of hypoxic tumors in situ.

## Data Availability

The data presented in this study are available on request from the corresponding author due to privacy.

## Supplementary Materials

The following supporting information can be downloaded at: https://www.mdpi.com/article/10.3390/ma17143495/s1, Figure S1: (a) Absorption curves of CSNP aqueous solution at different concentrations. (b) Linear standard curve of CSNP aqueous solution obtained from (a); Figure S2: (a) High-solution XPS spectrum of Si 2p for CSNP. (b) EDS characterization of CSNP. (c) Fluorescence spectrum of DCFH aqueous solution at 525 nm under 660 nm laser irradiation for ROS detection; Figure S3: (a) UV-vis-NIR diffuse reflectance spectrum of CSNP. (b) The Mott–Schottky plot of CSNP (the potential was measured against an Ag/AgCl reference and converted to NHE potentials using E_(NHE)_ = E_(Ag/AgCl)_ + 0.197 eV); Figure S4: (a) UV-vis-NIR spectra of TMB solutions in CSNP treated with H_2_O_2_ at different times. (b) UV-vis-NIR spectra and photographs of TMB aqueous solution at different times at pH = 6.5 in PBS with 1 mM H_2_O_2_ and 10 mM GSH. (c) Time-dependent degradation of DPBF caused by ^•^O_2_^−^ in hypoxia conditions under irradiation. (d) Time-dependent degradation of DPBF caused by ^•^O_2_^−^ in normoxia conditions under irradiation; Figure S5: Toxicity of H_2_O_2_-only treatment at different pH values and in the presence of laser-only treatment to CT26 cells; Figure S6: CT26 cells co-stained with Calcein-AM/PI under H_2_O_2_-only and laser-only treatments by CLSM. Scale bar: 50 μm; Figure S7: (a) Detection of ^•^O_2_^−^ in CT26 cells with DHE probe under H_2_O_2_-only conditions. (b) Fluorescence images of ROS production in CT26 cells stained with DCFH-DA (a green ROS probe) following H_2_O_2_-only and CSNP-only treatment. Scale bar: 50 μm; Figure S8: H&E staining photographs of main organs from mice injected with CSNP after 14 days. Scale bar: 100 μm.
