# Supplementary material for: A Copper Silicate-Based Multifunctional Nanoplatform with Glutathione Depletion and Hypoxia Relief for Synergistic Photodynamic/Chemodynamic Therapy
Source: Materials (Basel). 2024 Jul 15;17(14):3495. doi: 10.3390/ma17143495 (PMC11278046; doi:10.3390/ma17143495)
Supplement: Supplementary file 1 [file materials-17-03495-s001.zip › materials-3069238-supplementary.pdf]

A Copper Silicate-Based Multifunctional Nanoplatform with Glutathione Depletion and Hypoxia Relief for Synergistic Photodynamic/Chemodynamic Therapy

Meiqi Shao [1,2], Wei Zhang [3], Fu Wang [2], Lan Wang [3,*] and Hong Du [1,*]

[1] *Xinjiang Key Laboratory of Energy Storage and Photoelectrocatalytic Materials & Chemistry and Chemical Engineering, Xinjiang Normal University, Urumqi 830054, China*

[2] *Shenzhen Research Institute, Shanghai Jiao Tong University, Shenzhen 518057, China*

[3] *Environmental Science and Engineering, Shaanxi University of Science and Technology, Xi'an 710021, China*

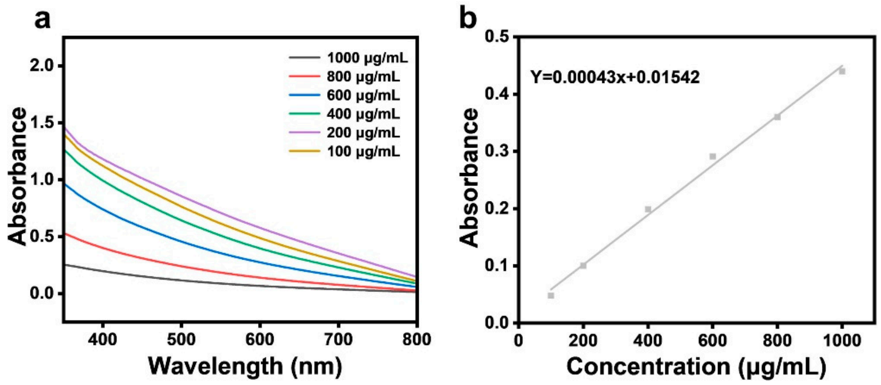

Figure S1. (**a**) Absorption curves of CSNP aqueous solution at different concentrations. (**b**) Linear standard curve of CSNP aqueous solution obtained from (a).

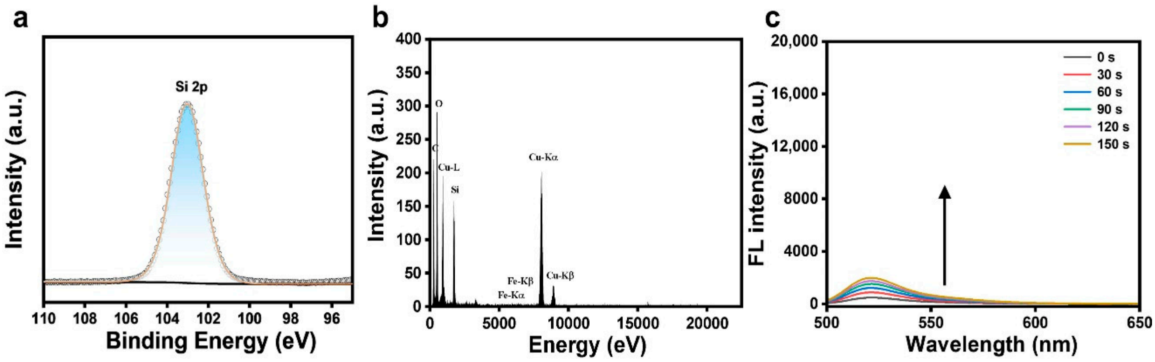

Figure S2. (**a**) High-solution XPS spectrum of Si 2p for CSNP. (**b**) EDS characterization of CSNP. (**c**) Fluorescence spectrum of DCFH aqueous solution at 525 nm under 660 nm laser irradiation for ROS detection.

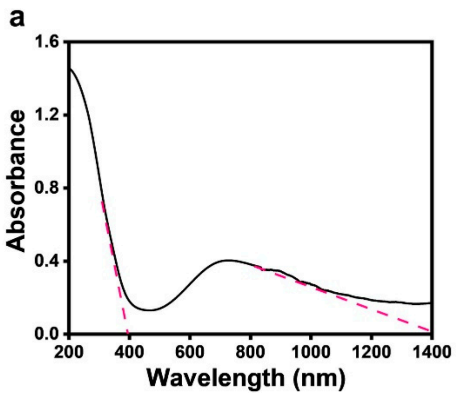

Figure S3. (**a**) UV-vis-NIR diffuse reflectance spectrum of CSNP. (**b**) The Mott–Schottky plot of CSNP (the potential was measured against an Ag/AgCl reference and converted to NHE potentials using $E_{(NHE)} = E_{(Ag/AgCl)} + 0.197$ eV).

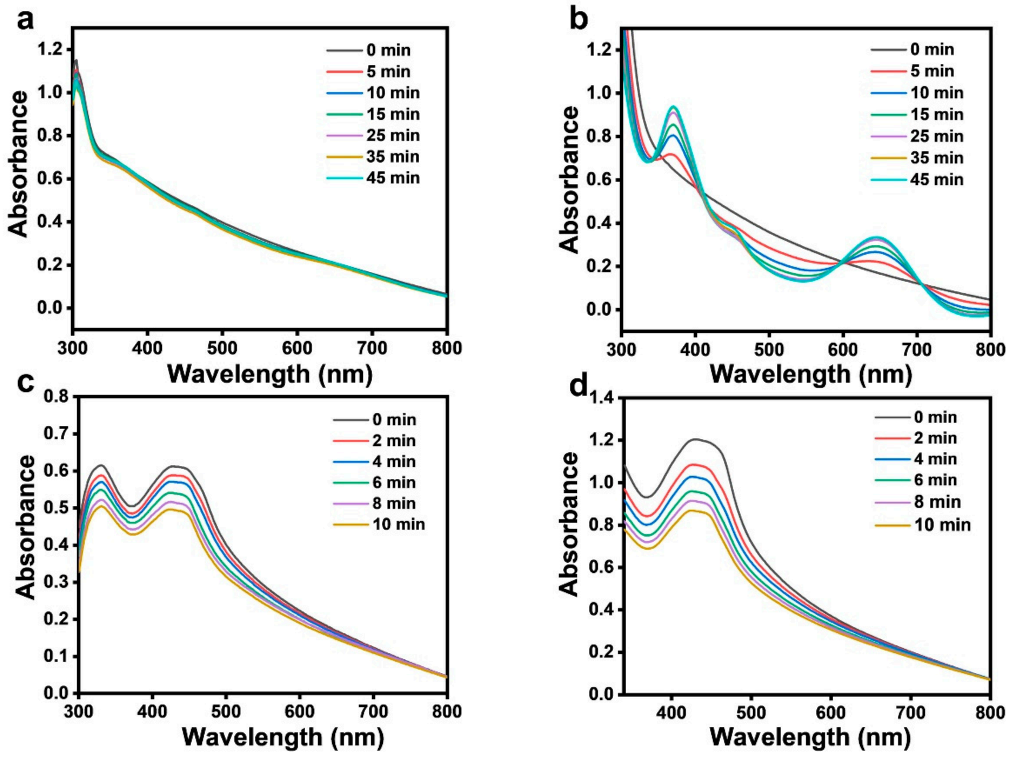

Figure S4. (**a**) UV-vis-NIR spectra of TMB solutions in CSNP treated with H_2O_2 at different times. (**b**) UV-vis-NIR spectra and photographs of TMB aqueous solution at different times at pH = 6.5 in PBS with 1 mM H_2O_2 and 10 mM GSH. (**c**) Time-dependent degradation of DPBF caused by $^{\bullet}O_2^-$ in hypoxia conditions under irradiation. (**d**) Time-dependent degradation of DPBF caused by $^{\bullet}O_2^-$ in normoxia conditions under irradiation.

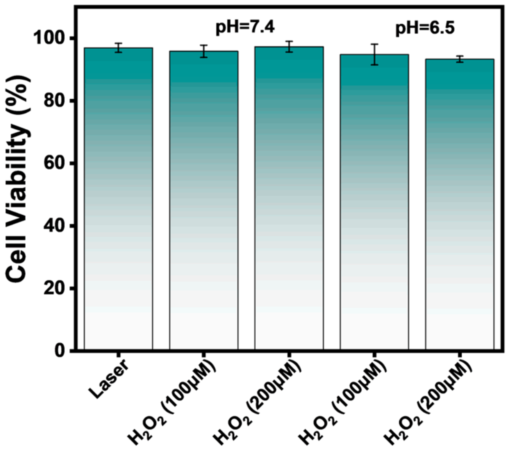

Figure S5. Toxicity of H_2O_2-only treatment at different pH values and in the presence of laser-only treatment to CT26 cells.

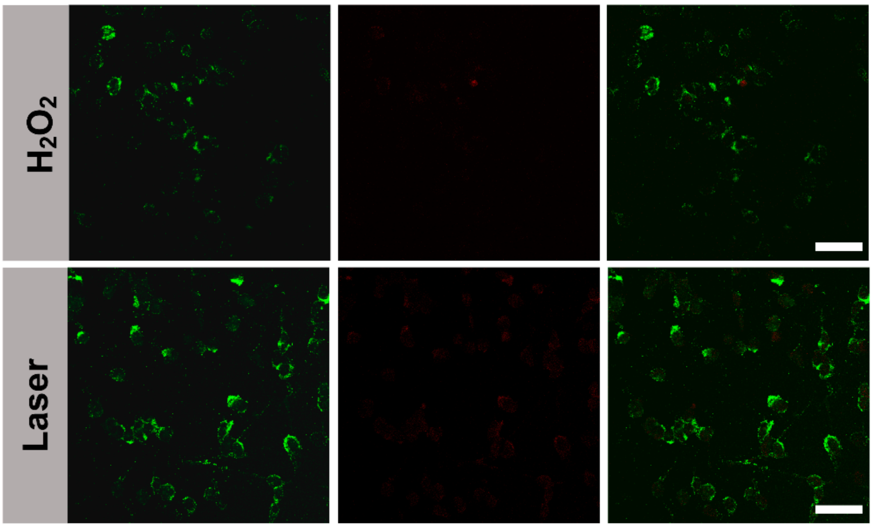

Figure S6. CT26 cells co-stained with Calcein-AM/PI under H_2O_2-only and laser-only treatments by CLSM. Scale bar: 50 μm.

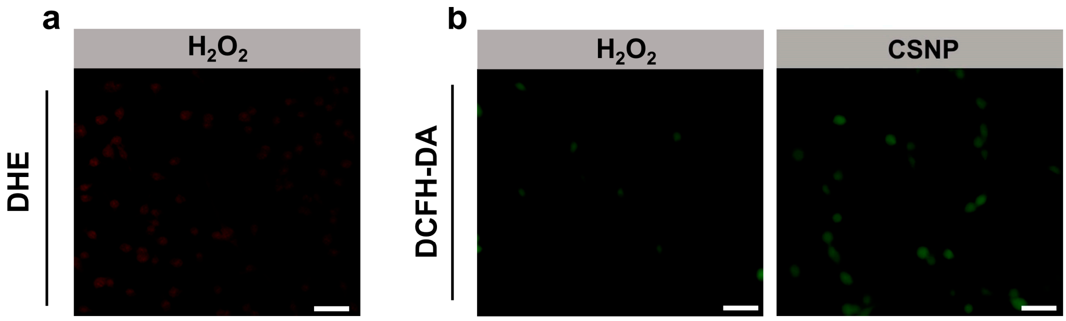

Figure S7. (a) Detection of $^{\bullet}O_2^-$ in CT26 cells with DHE probe under H_2O_2-only conditions. **(b)** Fluorescence images of ROS production in CT26 cells stained with DCFH-DA (a green ROS probe) following H_2O_2-only and CSNP-only treatment. Scale bar: 50 μm.

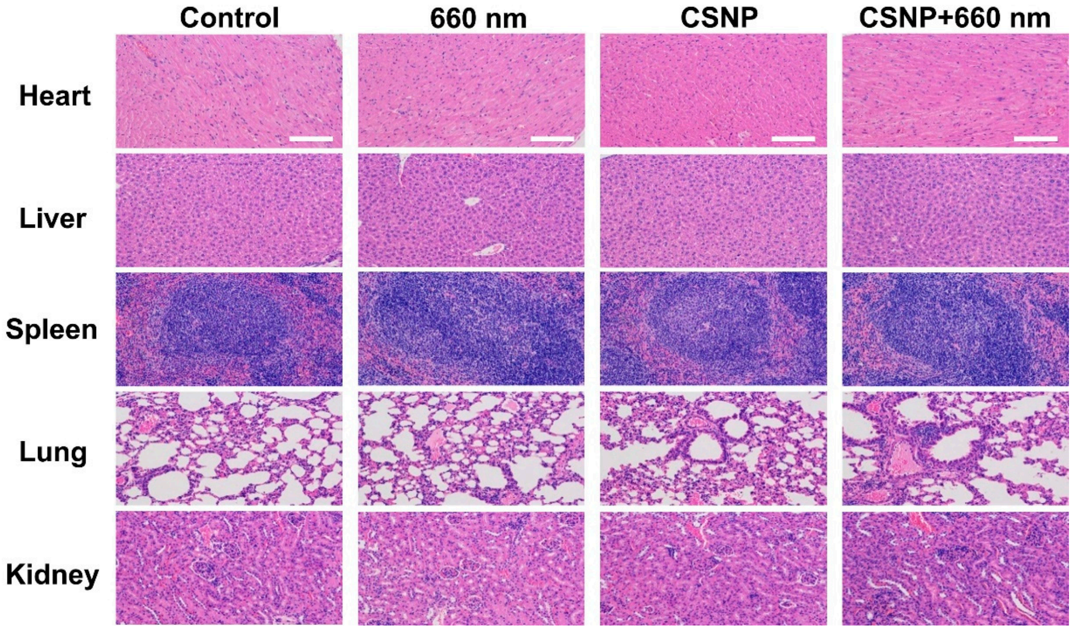

Figure S8. H&E staining photographs of main organs from mice injected with CSNP after 14 days. Scale bar: 100 μm.
